# Targeted delivery of immunogenic antigen to the tumor microenvironment after irradiation facilitates tumor control

**DOI:** 10.1186/2051-1426-1-S1-P78

**Published:** 2013-11-07

**Authors:** Tc Wu, Chien-Fu Hung

**Affiliations:** 1 Pathology, Johns Hopkins, Baltimore, MD, USA

## 

Tumor control by radiation therapy (RT) acts beyond direct RT-induced cell death and has the ability to stimulate a systemic anti-tumor immune response through antigen release and sterile inflammation. However, RT-induced myeloid cell recruitment can interfere with the anti-tumor immune response and facilitate tumor growth thus resulting in recurrence of the primary tumors to cause death in patients. In the present study, we used an HPV E7 expressing tumor model, TC-1, to test whether, by injecting E7 or ovalbumin antigenic peptide in the radiated tumor, we would be able to prime CD8+ T cells and load the myeloid cells with antigen, rendering them susceptible to killing by the antigen specific T cells. Here, we show that by combining RT and targeted delivery of antigenic peptide to the tumor, the adjuvant effect generated by RT itself is sufficient to elicit the priming and expansion of antigen-specific CD8+ T cells through the type 1 interferon and toll like receptor 4 dependent pathways. Furthermore, our approach generates potent therapeutic antitumor effects compared to RT or intratumor injection of peptide alone. In addition, we demonstrated that CTL-mediated killing of stromal cells, including CD11b+ myeloid cells, in the tumor by our approach is important for the control of tumor growth using two different transgenic mouse models. This approach represents a platform technology that can potentially be applied to many different tumors to generate a potent therapeutic antitumor effect.

